# BLU-DAT: a new reliable and accurate arthrometer for measuring anterior knee laxity

**DOI:** 10.1007/s00167-023-07534-5

**Published:** 2023-08-08

**Authors:** Alessandro Colosio, Alessandra Scaini, Marcello Motta, Marco Paderno, Maristella F. Saccomanno, Pierfrancesco Bettinsoli, Giovanni Bonaspetti, Giuseppe Milano

**Affiliations:** 1 Department of Medical and Surgical Specialties, Radiological Sciences, and Public Health, University of Brescia, Brescia, Italy; 2 Department of Bone and Joint Surgery, Spedali Civili, Brescia, Italy; 3 Department of Orthopaedics and Traumatology, Clinica S. Anna, Brescia, Italy; 4 ASST Spedali Civili, UOC Clinica Ortopedica, Piazzale Spedali Civili 1, 25123 Brescia, BS Italy

**Keywords:** Knee, Anterior cruciate ligament, Anterior tibial translation, Arthrometer

## Abstract

**Purpose:**

Although many arthrometers have been developed to assess anterior knee laxity, reliability and diagnostic accuracy of these devices are still debated. The aim of the present study was to evaluate the validity of a new arthrometer in the outpatient setting, with the hypothesis that it had good validity in terms of reliability and diagnostic accuracy.

**Methods:**

Seventy-eight subjects (39 with ACL injury and 39 with normal ACL) were tested. ATT was assessed by means of the Lachman test at 30° of flexion with a new testing device (BLU-DAT) under three different loading conditions: 7 kg (69 N), 9 kg (88 N) and maximum (MMT). The tests were performed on both knees to obtain SSD. In the ACL injury group, the tests were performed by two examiners and one of them repeated a second test series. Inter- and intra-observer reliability were assessed with the intraclass correlation coefficients (ICCs) for the average SSD measures. In the normal-ACL group, the analysis was performed with the same testing setup. Side-to-side difference measures of the two groups at every loading condition were compared by Student’s *t* test. Data of test series were dichotomized based on the threshold value of 3-mm SSD as pathological ATT and 2 × 2 contingency tables were used to assess diagnostic accuracy.

**Results:**

The ICCs for intra-observer reliability at 7-kg (69 N), 9-kg (88 N) and MMT measurements were 0.781, 0.855 and 0.913, respectively. The ICC for inter-observer reliability at 7-kg (69 N), 9-kg (88 N) and MMT measurements were 0.701, 0.845 and 0.834, respectively. Comparison between the two groups showed a significant mean difference ranging from 3.4 mm for 7-kg (69 N) load to 4.6 mm for MMT. Overall accuracy ranged from 84.6% for 7-kg load to 98.7% for MMT.

**Conclusion:**

The BLU-DAT has proven to be an instrument with good intra- and inter-observer reliability and very good accuracy in the diagnosis of ACL injuries in the outpatient setting. So, the BLU-DAT can be a new useful tool in everyday clinical practice to assist in the diagnosis of ACL injury.

**Level of evidence:**

II.

**Supplementary Information:**

The online version contains supplementary material available at 10.1007/s00167-023-07534-5.

## Introduction

Diagnosis of ACL injury has been greatly improved in the last decades, thanks to the development of imaging modalities for the diagnosis of musculoskeletal diseases. Nonetheless, it is still strictly based on the clinical examination and functional tests that measure knee laxity and by assessing the functional competence of ACL. Among various tests, the Lachman test and the pivot shift test are the most accurate for diagnosing ACL insufficiency, both in acute and chronic conditions [23].

Quantification of ATT has not only diagnostic value in ACL injuries but also has significance in the outcome assessment of reconstructive surgery [11]. For this reason, various devices called “arthrometers” have been developed since the 1980s. These include their progenitor KT 1000 (MEDmetric Corporation, San Diego, CA, USA), Rolimeter (Aircast, Summit, New Jersey, US), GNRB (Genurob, Laval, France) and many others. Numerous studies evaluated the reliability and accuracy of these devices, both in clinical and experimental settings. A recent consensus [22] has encouraged their use to increase the reliability and validity of the assessment of anterior knee laxity, especially for follow-up evaluation of surgical treatments.

Indeed, the reliability and diagnostic accuracy of knee arthrometers can be affected by several factors, such as knee position, examiner’s experience and patient’s compliance [19]. Besides, some devices are unsuitable for the outpatient setting because of their size or costs. An instrumented laxity testing device should handle all these issues to be as accurate and reliable as possible and suitable for every condition of use.

The purpose of the present study was to evaluate intra- and inter-observer reliability and diagnostic accuracy of a new portable device for testing ATT, which, unlike the devices already on the market, is suitable for both clinical and experimental settings. The hypothesis of the study was that the new arthrometer has good validity in terms of both reproducibility and diagnostic accuracy.

## Material and methods

The local IRB and Ethic Committee approved the study protocol (Prot. No. 4767, University of Brescia, ASST Spedali Civili). The study was conducted according to the principles of good clinical practice and of the Declaration of Helsinki and its updated version (Tokyo 2004). The study was designed as a prospective observational study with a control group. Guidelines established by the QAREL checklist for reliability study were followed [10]. Also, standards for reporting diagnostic accuracy studies (STARD) were adopted to assess diagnostic accuracy [3].

### Study population

All patients undergoing knee surgery for ACL reconstruction or other intraarticular surgical procedures (meniscal or cartilage treatments) were considered eligible for the study. Inclusion criteria were: age older than 18 years, and acceptance to enter the study. The patients were divided into two groups according to the presence of an ACL rupture or not. ACL injury group consisted of patients with clinically and MRI-confirmed ACL injury and scheduled for ACL reconstruction surgery. Control group consisted of subjects undergoing knee arthroscopy for other injuries (meniscal and/or cartilage), in which ACL integrity was documented preoperatively. In all cases, enrolment was confirmed at the time of arthroscopy when ACL status was definitively confirmed. Exclusion criteria were: osteoarthritis to one or both knees documented on preoperative radiographic examinations, history of trauma or previous surgery to the contralateral knee, and inflammatory or neurologic diseases (systemic or local).

### Testing device

Preoperative assessment of anterior knee laxity was performed using a new device for quantification of ATT (BLU-DAT; FGP srl, Dossobuono, VR, Italy).

**Fig. 1:**
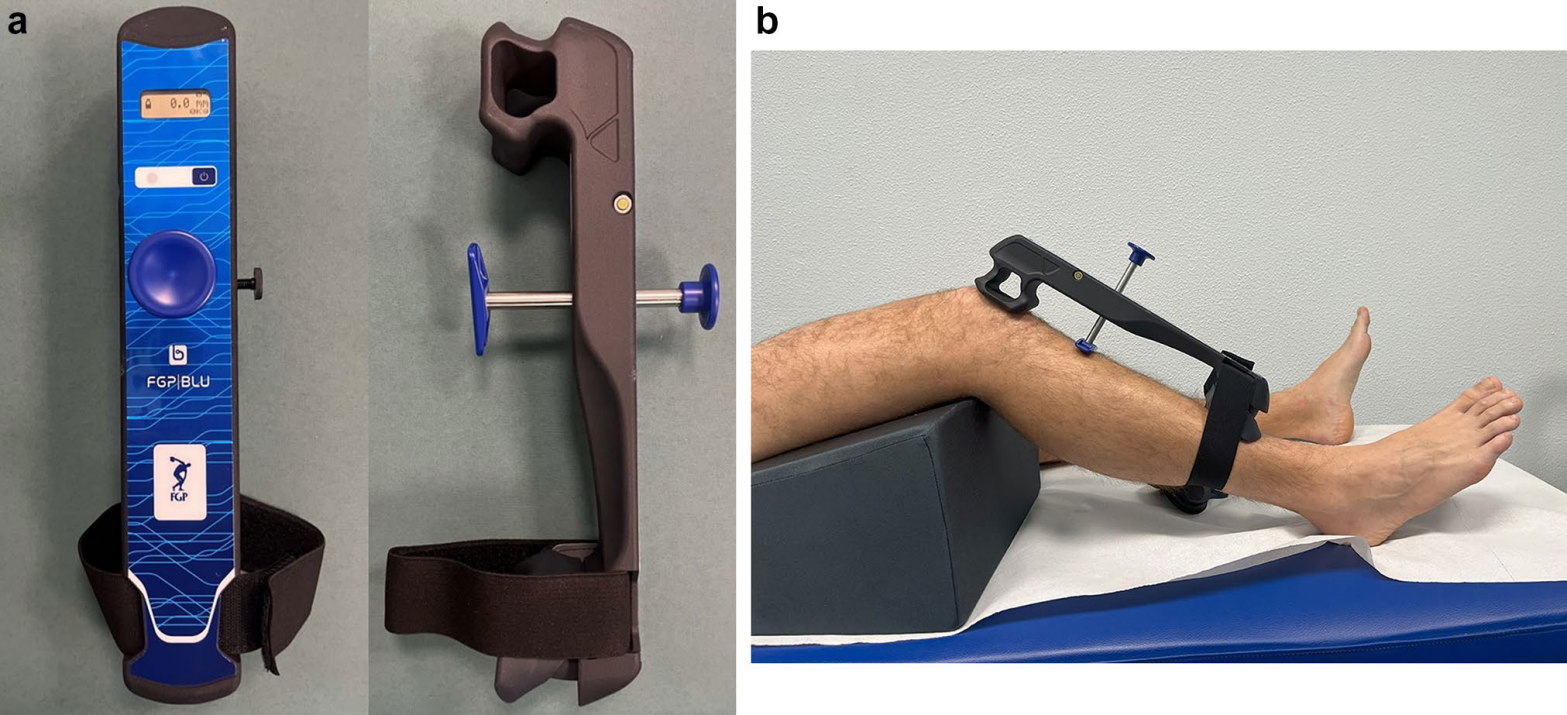
**A**The BLU-DAT laxity testing device. Displacement on the sagittal plane is measured by a magnetic linear encoder whose mobile part is applied to a sliding rod enveloped in a guiding probe, which is attached to the body of the arthrometer. **B** Correct position with the upper support positioned on the patella, the probe on the anterior face of the tibia, and the lower support at the level of the distal tibia, with the knee at 30° of flexion

**Fig. 2:**
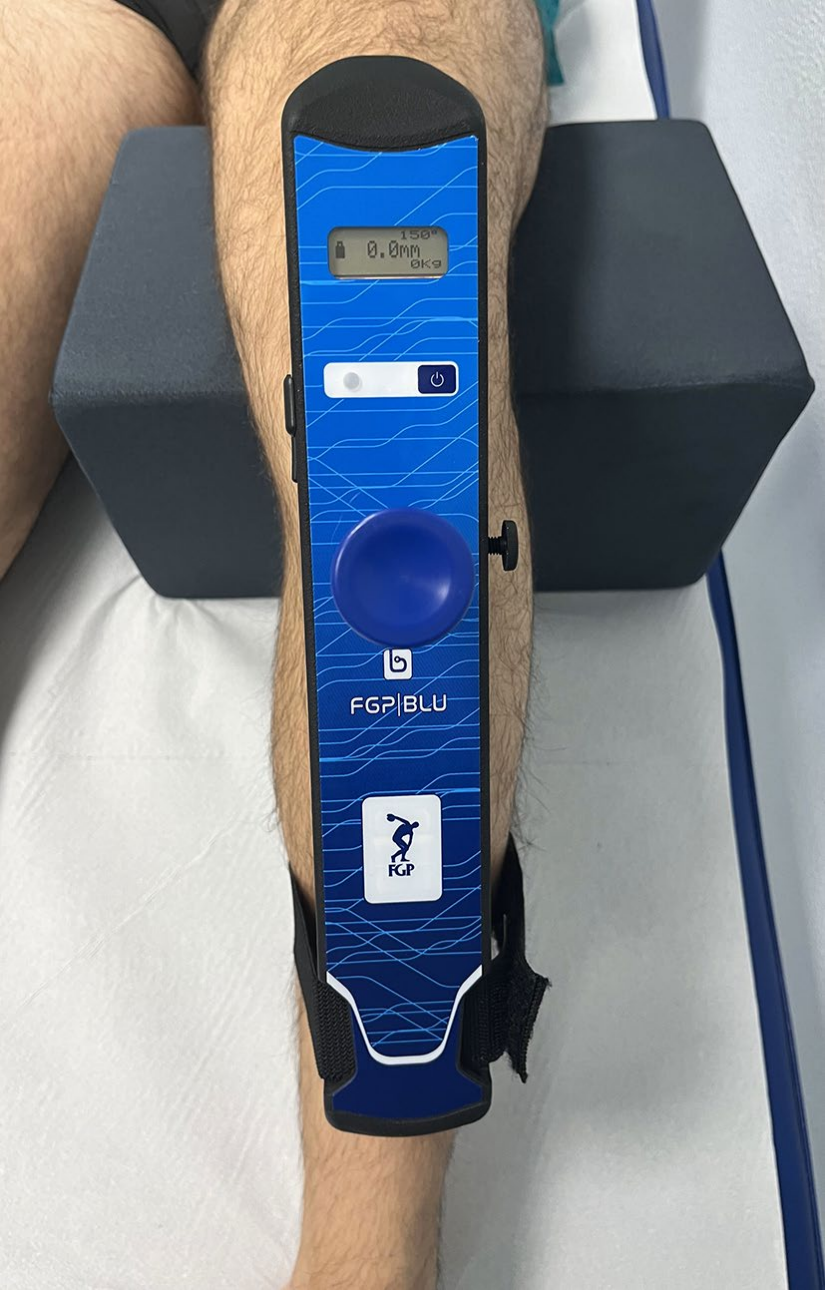
The examiner can control the variables during the examination on the digital display, where the knee flexion angle, anterior tibial translation expressed in mm and applied force expressed in kilograms can be viewed

The BLU-DAT testing device is designed to measure the anterior translation of the tibia respect to the femur on the sagittal plane. Displacement is measured by the mean of a magnetic linear encoder whose mobile part is applied to a sliding rod enveloped in a guide (the probe), whereas the feeler is fixed to the arthrometer body **(**Fig. 1**)**. Measurement of anterior tibial translation relative to the femur is shown on the device display. The resolution of the ATT measurement is 0.1 mm. The device is also equipped with sensors that evaluate the degree of knee flexion during the test, thus allowing one to check the proper knee flexion angle according to the clinical testing (i.e., Lachman test and anterior drawer test) (Fig. 2).

**Fig. 3:**
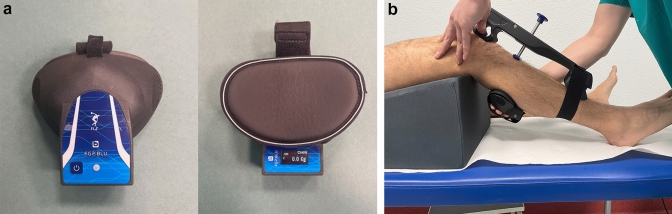
**A** The dynamometer, connected via Bluetooth to the system, allows the applied force to be recorded. **B** The dynamometer is placed on the examiner's hand applying anterior traction to the tibia

The arthrometer has two supports: the proximal one should be placed at the level of the patella, whereas the distal one on the distal tibia. The right location of the device is achieved by making the probe falling on the anterior aspect of the proximal tibia. The system can be connected by Bluetooth to an accessory dynamometer that allows to quantify the applied force (Fig. 3). This extension allows to combine displacement data to the force applied while performing the test.

### Evaluation

All patients underwent a preoperative assessment of anterior knee laxity by measuring ATT with BLU-DAT. The Lachman test was used to assess ATT, as it has proven to have high diagnostic accuracy [7]. Measurements in the ACL-injured group were performed by two investigators: a sports-medicine experienced orthopaedic surgeon (examiner A) and (examiner B). The examiner B repeated the measurements on the ACL-injured group after three weeks and acquired measurements in the control group. Both examiners were trained in the use of the device before the start of the trial.

All measurements were performed in a standardized setting, with a knee at 30 degrees of flexion in neutral rotation with the aid of a semi-rigid wedge placed at the level of the popliteal fossa. Once the arthrometer had been correctly applied, as previously described, the display was reset (ATT = 0 mm) and the Lachman test was performed. Lachman test was performed at three different loading conditions: 7 kg (69 N), 9 kg (88N), and MMT. Traction forces were chosen based on prior validation studies for knee arthrometers [1].

Three measurements of ATT, expressed in millimeters (mm), were collected for each measurement series and at every loading condition on the affected and contralateral healthy knee in both groups. The mean value of three consecutive measurements was calculated for each measurement series and ATT was expressed as a difference in mm between the mean ATT of the affected and contralateral knee (side-to-side difference).

The order in which the patients were assessed by the two examiners at first evaluation was varied according to a randomized sequence to limit the risk of assessment bias due to the effect of the potential reduction in patient compliance during the subsequent series of instrumental assessments related to the potential different skill of the examiners (examiner bias) [18]. Each investigator was blinded to the results obtained by the other investigator.

### Outcome measures

The primary outcomes of the study were the intra- and inter-observer reliability of the ATT measurements in the ACL-injured subjects. Reliability measurements for every loading condition were expressed by the intraclass correlation coefficients (ICCs). The ICC values vary between 0 and 1 (assuming perfect reliability for values of 1) and reliability was interpreted as follows: ICC < 0.5 = poor, ICC between 0.50 and 0.75 = moderate, ICC between 0.75 and 0.90 = good and ICC > 0.9 = excellent.

Secondary outcome of the study was diagnostic accuracy. We estimated the ability of the instrument to detect a difference in ATT between the two groups (ACL-injured and healthy knees). The threshold of 3 mm of side-to-side difference in ATT was considered pathologic and diagnostic for an ACL rupture [16, 24].

### Statistical analysis

Data were analyzed using statistical software (IBM SPSS Statistics 25; IBM, Armonk, NY, USA). For the measurement of intra-observer reliability, a two-way mixed-effects model of ICC for average measures (*k* = 2) and absolute agreement was used. For inter-observer reliability, we calculated ICC using a one-way random effect model for average measures (*k* = 2) and absolute agreement (ICC 1,2) [13]. In addition, from the obtained ICC and standard deviation of each measurement series, the accuracy of the measurements was established by calculating the SEM between the observations.

Diagnostic accuracy was assessed by comparing the side-to-side differences of the two groups at every loading condition by means of Student’s *t* test. Then, data of the two groups were dichotomized based on the threshold value of 3 mm as pathological ATT (positive test) and 2 × 2 contingency tables were used to assess diagnostic accuracy in terms of sensitivity, specificity, NLR, PLR, PPV, NPV, AUC, DOR, Youden index, and overall accuracy. Statistical significance was considered for *p* values < 0.05. Ninety-five percent confidence intervals were provided for each statistical test. Sample size was calculated based on the primary outcomes of the study (intra- and inter-observer reliability) and established in accordance with Walter et al. [26] for reliability studies by calculation of the ICC based on two observations. A sample size of 39 cases was appropriate based on a reliability coefficient equal to 0.5 for the null hypothesis (R0) and equal to 0.7 for the alternative hypothesis (R1), given α equal to 0.05 and a power (1 − β) equal to 0.80 [2].

Sample size was confirmed to be adequate also for the secondary outcome (diagnostic accuracy). Based on a pilot sample of the first 20 cases analyzed with a definite diagnosis of ACL injury, the mean side-to-side difference value for ATT observed was 4.34 ± 5.24 mm. Given a minimal clinically important difference equal or greater than 3 mm between the ACL-injured group and the control group [16, 24], an effect size of 0.57 and a sample size of 39 cases per group was obtained based on a two-way alternative hypothesis, given a value of α equal to 0.05 and a power (1 − β) equal to 0.80.

## Results

**Fig. 4:**
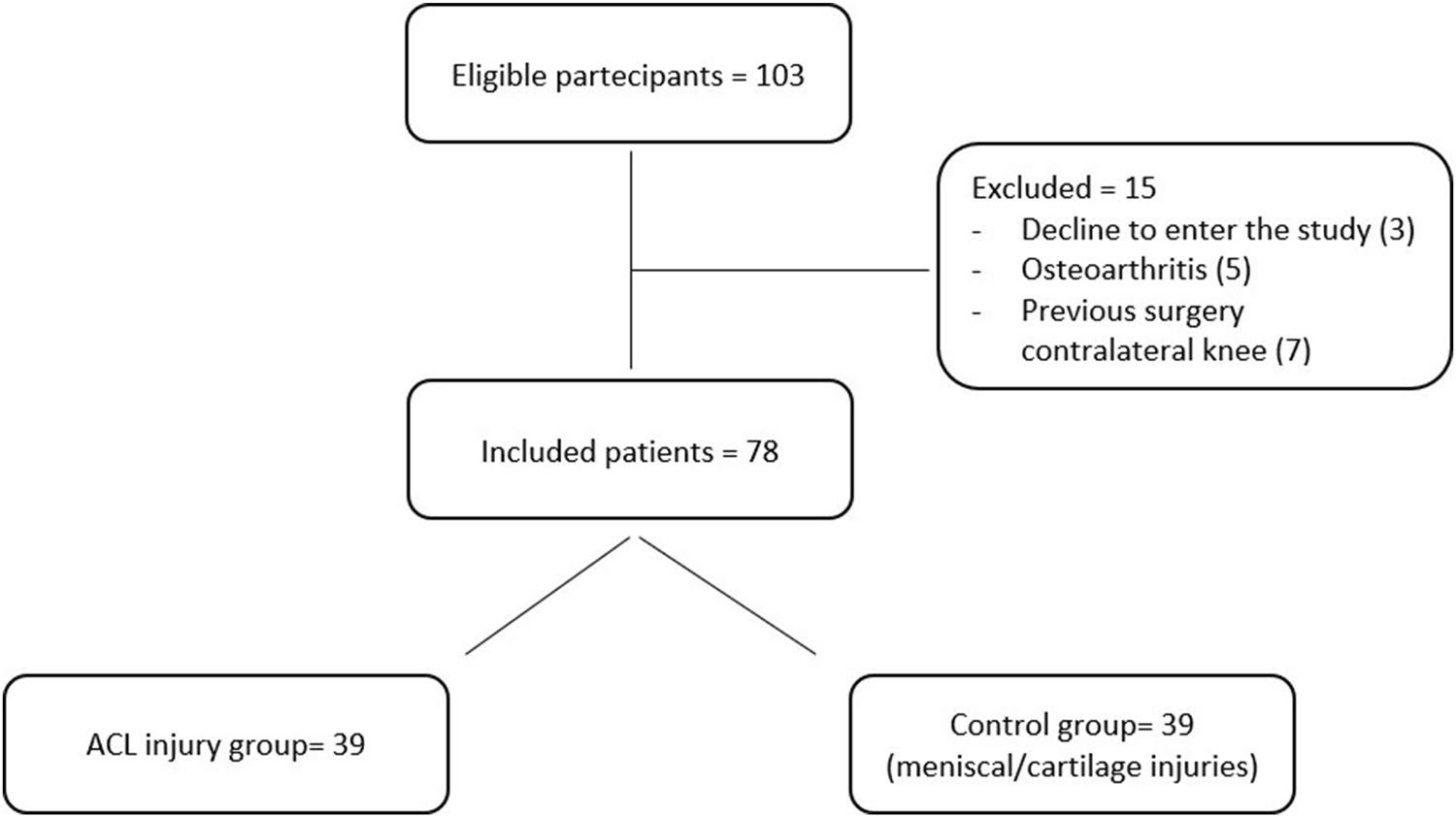
Study flowchart

Seventy-eight subjects were consecutively recruited for the present study and divided into two groups of 39 patients each (Fig. 4). ACL-injury group consisted of 33 males and 6 females (mean age: 29.8 ± 13.2 years); the control group consisted of 25 males and 14 females (mean age: 38.3 ± 11.9 years).

Descriptive data for all measurement series on ACL-injured patients are reported in Table 1.

**Table 1:** Descriptive data for all measurement series on ACL-injured patients

Rater	Measurement	Min	Max	Mean	SD
Rater 1 Observation 1	7 kg (69 N)	2.2	6	3.4	0.8
	9 kg (88 N)	2.1	6.2	4	0.9
	MMT	2.1	8	4.8	1.2
Rater 1 Observation 2	7 kg (69 N)	2.2	5.5	3.5	0.9
	9 kg (88 N)	2.1	8.2	4.2	1.3
	MMT	2.1	8.6	5	1.3
Rater 2	7 kg (69 N)	2.1	6.8	3.6	1.1
	9 kg (88 N)	2.5	7	4.1	1.1
	MMT	3.1	8.1	4.9	1

Intra-observer reliability was good for 7-kg and 9-kg measurements and excellent for MMT. Inter-observer reliability was moderate for 7-kg measurements ang good for 9-kg and MMT. Intraclass correlation coefficients for intra- and inter-observer reliability are shown in Table 2. SEMs are reported in Table 3.

**Table 2:** Intraclass correlation coefficients for intra- and inter- observer reliability

Outcome measure	ICC	95% CI	
		Lower limit	Upper limit
**Intra-observer reliability**			
7 kg (69 N)	0.781	0.583	0.885
9 kg (88 N)	0.855	0.723	0.924
MMT	0.913	0.836	0.954
**Inter-observer reliability**			
7 kg (69 N)	0.701	0.435	0.842
9 kg (88 N)	0.845	0.707	0.919
MMT	0.834	0.685	0.913

**Table 3:** Standard error of measurements for intra- and inter-observer reliability

Outcome measure	SEM
**Intra-observer reliability**	
7 kg (69 N)	0.44
9 kg (88 N)	0.42
MMT	0.35
**Inter-observer reliability**	
7 kg (69 N)	0.51
9 kg (88 N)	0.44
MMT	0.48

Comparison between ACL-injured and control groups for the side-to-side difference in ATT showed a significant difference between the two groups at every loading condition. Mean difference between groups ranged from 3.38 mm for 7-kg measurement to 4.55 mm for MMT (Table 4).

**Table 4:** Comparison between groups for anterior tibial translation (mean ± SD) at different loading conditions

Measurement	ACL injury (*N* = 39)	Normal (*N* = 39)	*p*	Difference between means	95% CI	
					Lower limit	Upper limit
7 kg (69 N)	3.4 ± 0.85	0 ± 0.76	< 0.0001	3.4	3.0	3.8
9 kg (88 N)	4.0 ± 0.95	0.3 ± 0.72	< 0.0001	3.7	3.3	4.1
MMT	4.8 ± 1.16	0.3 ± 0.89	< 0.0001	4.6	4.1	5.0

Output of diagnostic accuracy estimates is reported in Table 5. As no false positives were reported at all loading conditions, PLR, AUC, DOR and Youden index could not be calculated. Overall accuracy ranged from 84.6% for 7-kg measurements to 98.7% for MMT. Detailed diagnostic accuracy measures are reported in Table 6.

**Table 5:** Contingency table for diagnostic accuracy of ATT at different loading conditions

ATT	7 kg (69 N)		9 kg (88 N)		MMT	
	ACL injury (*N* = 39)	Normal (*N* = 39)	ACL injury (*N* = 39)	Normal (*N* = 39)	ACL injury (*N* = 39)	Normal (*N* = 39)
≥ 3 mm	27 (69.2%)	0	34 (87.2%)	0	38 (97.4%)	0
< 3 mm	12 (30.8%)	39 (100%)	5 (12.8%)	39 (100%)	1 (2.6%)	39 (100%)

**Table 6:** Diagnostic accuracy measures for ATT at different loading conditions

Statistics	7 kg (69 N)			9 kg (88 N)			MMT		
	Value	95% CI		Value	95% CI		Value	95% CI	
		Lower limit	Upper limit		Lower limit	Upper limit		Lower limit	Upper limit
Sensitivity	69.2%	52	83	87.2%	72.6	95.7	97.4%	86.5	99.9
Specificity	100%	91.0	100	100%	91.0	100	100%	91.0	100
Negative likelihood ratio (NLR)	0.3	0.2	0.5	0.13	0.1	0.3	0.03	0	0.2
Positive predicitive value (PPV)	100%	–	–	100%	–	–	100%	–	–
Negative predicitive value (NPV)	76.5%	67.0	83.9	88.6%	77.5	94.7	97.5%	84.9	99.6
Accuracy	84.6%	74.7	91.8	93.6%	85.7	97.9	98.7%	93.1	99.9

## Discussion

The main findings of the present study are that the BLU-DAT has proven to be a valid instrument for testing anterior knee laxity and that the greater were loading conditions the better were results in terms of intra- and inter-observer reliability for measuring ATT.

Since the introduction of the KT-1000, the progenitor of arthrometers, numerous studies analyzed the reliability of these instruments to reproducibly diagnose ACL injury. KT-1000 and KT-2000, its successor, were certainly the most analyzed. The comparison of reliability results is not easy, as there are numerous studies in the literature that reported discordant reliability data, from poor to excellent, especially for inter-observer reliability with ICCs ranging from 0.41 to 0.92 [20, 27], but also often used different reliability assessment methods.

A recent study by Runer et al. [17] showed that the reproducibility analysis of different arthrometers (KT-1000, Rolimeter, KLT and Kira) was satisfactory for intra-observer reliability, but unsatisfactory for inter-observer reliability, thus concluding that anterior laxity analysis is best analyzed by the same operator. In another study, Murgier et al. [15] analyzed the agreement of measurements between different arthrometers (KT-1000, Rolimeter, GNRB and Telos) and found that different devices are difficult to compare in terms of side-to-side difference.

As mentioned above, there are several confounding factors that can undermine the reliability of these devices. These include the type of test used, experience of the examiner, applied force, knee flexion angle [9], tibial rotation and patient’s compliance. Those limitations led to create devices that reduce the role of the examiner, like the GNRB, whose reproducibility and validity has been tested with optimal results [4, 12]. This automated arthrometer exerts via a linear jack thrust forces chosen by the examinator with the lower limb positioned in a rigid leg support with the knee at 0° of rotation. In addition, surface electrodes are applied to the back of the thigh to control hamstring relaxation of the tested knee (feedback effect). Several studies in the past have evaluated its reproducibility, the most recent evaluation was performed by Smith et al.[21] who found moderate to good intra-rater reliability (ICC = 0.72–0.83) and good inter-rater reliability (ICC = 0.76–0.81). Unfortunately, automation required to minimize the role of the confounding factors results in a loss of handling and an increase in costs, making it difficult for routine outpatient use, and therefore, not comparable to portable devices like Rolimeter. Validity of Rolimeter was tested in several studies [5, 17]. Hatcher et al. [6], in a setting very similar to that of our study, analyzed its reliability and reported excellent intra- and inter-observer reliability (ICC = 0.912 and 0.945, respectively) during the Lachman test at 30° of knee flexion. However, extreme handling can prevent control of some variables involved and can impair the standardization of the testing setup.

The BLU-DAT can be a good compromise between manageable arthrometers like Rolimeter, and more complex and expensive devices such as GNRB and KT-1000, being able to keep excellent handiness and maintaining an objective control of the knee flexion and of the applied force, thus allowing standardization of the test also in the routine outpatient activity. To confirm this, the evaluation of its reliability in a cadaver study was recently published [14]. Specifically, inter-rater reliability was evaluated under the same loading conditions as in the present study with ICC for average measurements very good at all different loads (0.89, 0.85 and 0.88 at 7 kg, 9 kg and MMT, respectively). In addition, agreement of the BLU-DAT with a gold standard such as stress radiographs analyzed with the Bland–Altman method showed good agreement with a mean difference between the two methods of 0.83 mm ± 2.1 mm (95% CI 0.55–1.11).

The second outcome analyzed in our study was diagnostic accuracy. The BLU-DAT proved to be very good in terms of accuracy for diagnosis of ACL rupture, with a diagnostic accuracy of 98.7%. Also, for this outcome, the best results were observed when performing the test at MMT.

This agrees with previous results reported with other devices. In a meta-analysis by van Eck et al. [25], several arthrometers (KT 1000, Stryker Knee Laxity Tester and Genucom Knee Analysis System) showed better sensitivity, specificity and overall accuracy at MMT than at lower loads.

A similar finding was found by Klouche et al. [8] with GNRB, where accuracy was proportional to the force applied, except for the maximum force they applied (250 N) and, as explained by the authors, this may be caused by the patients exceeding their pain threshold. In our test series, this type of reduction in compliance due to excessive traction never occurred.

In terms of diagnostic accuracy, a new automatic knee arthrometer (AKA) recently introduced by Niu et al. [16] is noteworthy, which allows minimizing the examiner's bias by means of an automatically performed thrust. The authors found higher accuracy than KT 2000 (sensitivity: 86% vs 83%; specificity: 95.5% vs 88.5%), using a threshold of 3 mm as in the study. Wu et al. [28], recently tested a new automatic knee arthrometer (Ligs Innomotion). At a load of 150 N (comparable to the MMT in the present study), the authors found the maximum AUC (0.857) and a sensitivity and specificity of 0.87 and 0.73, respectively. The present study has some limitations. First, the study lacks a direct comparison with another arthrometer that has already been validated and studied. Second, we did not assess ATT in a postoperative setting, therefore external validity of the study cannot be extended to the use of the device for assessment of surgical treatment at clinical follow-up. Finally, the investigators were blinded to the measurements of the other investigator, but for practical matters, they were aware of the group to which the individual patients belonged. The Blu-DAT can be routinely used in the outpatient setting to confirm the suspicion of an ACL tear.

## Conclusions

The BLU-DAT has proven to be an instrument with good intra- and inter-observer reliability and very good accuracy in the diagnosis of ACL injuries in the outpatient setting. MMT loading condition provided the best reliability and accuracy.

### Supplementary Information

Below is the link to the electronic supplementary material.

Supplementary file1 (DOCX 13 KB)

Supplementary file2 (SPS 2 KB)

## Abbreviations

ACL: Anterior cruciate ligament
ATT: Anterior tibial translation
MMT: Maximum manual traction
SSD: Side-to-side difference
ICC: Intraclass correlation coefficient
PLR: Positive likelihood ratio
NLR: Negative likelihood ratio
PPV: Positive predictive value
NPV: Negative predictive value
AUC: Area under the curve
DOR: Diagnostic odds ratio
SEM: Standard error of measurement
