# Supplementary material for: BLU-DAT: a new reliable and accurate arthrometer for measuring anterior knee laxity
Source: Knee Surg Sports Traumatol Arthrosc. 2023 Aug 8;31(11):4782–90. doi: 10.1007/s00167-023-07534-5 (PMC10598137; doi:10.1007/s00167-023-07534-5)
Supplement: Supplementary file 1 — Supplementary file1 (DOCX 13 KB) [file 167_2023_7534_MOESM1_ESM.docx]

MEANS TABLES=age lbs15rate1 lbs20rate1 mmtrate1 lbs15rate1bis lbs20rate1bis mmtrate1bis lbs15rate2

lbs20rate2 mmtrate2 BY group

/CELLS=MEAN COUNT STDDEV MIN MAX.

CROSSTABS

/TABLES=sex BY group

/FORMAT=AVALUE TABLES

/STATISTICS=CHISQ

/CELLS=COUNT COLUMN

/COUNT ROUND CELL.

T-TEST GROUPS=group(0 1)

/MISSING=ANALYSIS

/VARIABLES=lbs15rate1 lbs20rate1 mmtrate1 lbs15rate1bis lbs20rate1bis mmtrate1bis lbs15rate2

lbs20rate2 mmtrate2

/CRITERIA=CI(.95).

RELIABILITY

/VARIABLES=lbs15rate1 lbs15rate1bis

/SCALE('ALL VARIABLES') ALL

/MODEL=ALPHA

/ICC=MODEL(MIXED) TYPE(ABSOLUTE) CIN=95 TESTVAL=0.

RELIABILITY

/VARIABLES=lbs20rate1 lbs20rate1bis

/SCALE('ALL VARIABLES') ALL

/MODEL=ALPHA

/ICC=MODEL(MIXED) TYPE(ABSOLUTE) CIN=95 TESTVAL=0.

RELIABILITY

/VARIABLES=mmtrate1 mmtrate1bis

/SCALE('ALL VARIABLES') ALL

/MODEL=ALPHA

/ICC=MODEL(MIXED) TYPE(ABSOLUTE) CIN=95 TESTVAL=0.

RELIABILITY

/VARIABLES=lbs15rate1 lbs15rate2

/SCALE('ALL VARIABLES') ALL

/MODEL=ALPHA

/ICC=MODEL(MIXED) TYPE(ABSOLUTE) CIN=95 TESTVAL=0.

RELIABILITY

/VARIABLES=lbs20rate1 lbs20rate2

/SCALE('ALL VARIABLES') ALL

/MODEL=ALPHA

/ICC=MODEL(MIXED) TYPE(ABSOLUTE) CIN=95 TESTVAL=0.

RELIABILITY

/VARIABLES=mmtrate1 mmtrate2

/SCALE('ALL VARIABLES') ALL

/MODEL=ALPHA

/ICC=MODEL(MIXED) TYPE(ABSOLUTE) CIN=95 TESTVAL=0.

CROSSTABS

/TABLES=diagn7 BY group

/FORMAT=AVALUE TABLES

/STATISTICS=CHISQ

/CELLS=COUNT COLUMN

/COUNT ROUND CELL.

CROSSTABS

/TABLES=diagn9 BY group

/FORMAT=AVALUE TABLES

/STATISTICS=CHISQ

/CELLS=COUNT COLUMN

/COUNT ROUND CELL.

CROSSTABS

/TABLES=diagnmmt BY group

/FORMAT=AVALUE TABLES

/STATISTICS=CHISQ

/CELLS=COUNT COLUMN

/COUNT ROUND CELL.
